# Morphological and Immunopathological Aspects of Lingual Tissues in COVID-19

**DOI:** 10.3390/cells11071248

**Published:** 2022-04-06

**Authors:** Dolaji Henin, Gaia Pellegrini, Daniela Carmagnola, Giuseppe Carlo Lanza Attisano, Gianluca Lopez, Stefano Ferrero, Antonella Amendola, Danilo De Angelis, Elisabetta Tanzi, Claudia Dellavia

**Affiliations:** 1 Department of Biomedical, Surgical and Dental Sciences, Università degli Studi di Milano, Via Mangiagalli 31, 20133 Milan, Italy; dolaji.henin@unimi.it (D.H.); gaia.pellegrini@unimi.it (G.P.); stefano.ferrero@unimi.it (S.F.); claudia.dellavia@unimi.it (C.D.); 2 Laboratory of Forensic Anthropology and Odontology (LABANOF), Department of Biomedical Sciences for Health, Università degli Studi di Milano, Via Ponzio 7, 20133 Milan, Italy; giuseppe.lanzaattisano@gmail.com (G.C.L.A.); danilo.deangelis@unimi.it (D.D.A.); 3 Division of Pathology, Fondazione IRCCS Ca’ Granda Ospedale Maggiore Policlinico, Via Francesco Sforza 35, 20122 Milan, Italy; gianluca.lopez@unimi.it; 4 Department of Health Sciences, Università degli Studi di Milano, Via Pascal 36, 20133 Milan, Italy; antonella.amendola@unimi.it (A.A.); elisabetta.tanzi@unimi.it (E.T.)

**Keywords:** SARS-CoV-2, tongue, lingual papillae, taste buds

## Abstract

COVID-19, a recently emerged disease caused by SARS-CoV-2 infection, can present with different degrees of severity and a large variety of signs and symptoms. The oral manifestations of COVID-19 often involve the tongue, with loss of taste being one of the most common symptoms of the disease. This study aimed to detect SARS-CoV-2 RNA and assess possible morphological and immunopathological alterations in the lingual tissue of patients who died with a history of SARS-CoV-2 infection. Sixteen cadavers from 8 SARS-CoV-2 positive (COVID-19+) and 8 negative (COVID-19−) subjects provided 16 tongues, that were biopsied. Samples underwent molecular analysis through Real-Time RT-PCR for the detection of SARS-CoV-2 RNA. Lingual papillae were harvested and processed for histological analysis and for immunohistochemical evaluation for ACE2, IFN-γ and factor VIII. Real-Time RT-PCR revealed the presence of SARS-CoV-2 RNA in filiform, foliate, and circumvallate papillae in 6 out of 8 COVID-19+ subjects while all COVID-19− samples resulted negative. Histology showed a severe inflammation of COVID-19+ papillae with destruction of the taste buds. ACE2 and IFN-γ resulted downregulated in COVID-19+ and no differences were evidenced for factor VIII between the two groups. The virus was detectable in most COVID-19+ tongues. An inflammatory damage to the lingual papillae, putatively mediated by ACE2 and IFN-γ in tongues from COVID-19+ cadavers, was observed. Further investigations are needed to confirm these findings and deepen the association between taste disorders and inflammation in SARS-CoV-2 infection.

## 1. Introduction

COVID-19, a recently emerged disease caused by SARS-CoV-2 infection, can present with different degrees of severity and a large variety of signs and symptoms, including taste and smell dysfunction, that are quite specific for this condition [1,2]. The change in taste could be temporary or in some cases chronic. Wu et al. reported a 43% prevalence of gustatory disorders in patients with COVID-19 and a study by Nouchi et al. reported that 29% of the patients with olfactory or gustatory disorders develop a persistent form of impairment in the subsequent months. SARS-CoV-2 infection has also been associated with hyposalivation and xerostomia that could worsen taste perception [3,4,5].

COVID-19 may display other signs concerning the oral cavity beyond taste disfunction, such as scattered reddish macules of the tongue, ulcers, or painful inflammation, as reported by some authors [6]. The tongue is also known to be involved in various alterations associated with local and systemic diseases [7] with consequences on important functions for humans, such as phonation, deglutition, touch, and taste.

Non-gustatory and gustatory papillae are located on the tongue dorsum. Non-gustatory papillae are known as filiform papillae (FLP), are larger in number than gustatory papillae, are characterized by a superficial layer of cornified epithelium with an underlying connective axis, and have mainly mechanical and tactile functions.

The gustatory papillae can be distinguished in foliate (FP), circumvallate (VP), and fungiform papillae (FGP). In the depth of the epithelial layer, the gustatory papillae host taste buds (TB) characterized by specific sensory cells that transmit taste sensitivity.

TB are bulb-shaped structures composed of four cell types with different functions: dark-toned cells (type I cells, 50–70%) that are glia-like cells with secretory and phagocytotic functions, light-toned cells (type II cells, 15–30%) that sense the taste stimuli, taste cells (type III, 5–15%) that transmit taste signals to sensory afferent fibers, and few basal cells at the base of the structure (type IV) [8,9,10]. When a substance encounters the sensory cells, their receptors, innervated by afferent neurons, transduce the different stimuli via specific mechanisms.

Probably due to the complexity and redundancy of the taste system, taste disorders were usually uncommon, until the COVID-19 era. Their association to COVID-19 has been reported as a distinct symptom since the first weeks of the pandemic but, up to now, the pathogenesis of COVID-19 gustatory disorders has not been established with precision and unanimousness [10,11]. COVID-19 taste disorders coexist frequently with smell disorders. Anatomopathological studies on humans aiming to investigate smell disorders have revealed a destruction of the receptors at the level of the olfactory epithelium, but no similar findings were reported concerning TB [12,13,14]. Some authors have suggested that taste disorders might be a consequence of the loss of smell [15], while others hypothesize that the two symptoms are independent [16,17]. Investigating the histopathological characteristics of the tongue, and in particular of its TB in COVID-19 subjects, might be useful to provide further understanding of the mechanisms behind taste alterations.

SARS-CoV-2 is known to be present in the oral cavity during infection; in particular, it is harbored by saliva, largely used for the molecular diagnosis of COVID-19 [18,19]. SARS-CoV-2 enters the cell through ACE2, the angiotensin-converting enzyme 2, a membrane-bound protein expressed on the surface of a number of cells, such as endothelial cells [20] and epithelia of the upper aero-digestive tract, including the oral cavity [21]. In the latter, ACE2 was mapped and has been shown to be strongly expressed in the lingual dorsum, in the stratum granulosum of the epithelial layer of the lingual papillae, and in in type II cells of TB [22]. After the virus enters the cell, the host antiviral defense in humans acts through chemokines, cytokines, and through interferon (IFN), as already reported in other inflammatory diseases. In particular, serum levels of IFN-γ in SARS-CoV-2 infected patients are elevated due to activation of Th1 and Th2 cells [23], and this contributes to different aspects of COVID-19 pathogenesis [24]. Some authors have reported that IFN-γ inflammation triggered by several factors could lead to taste function alteration [25,26]. The inflammation cascade in SARS-CoV-2 infection involves also thrombo-inflammation, with an increased level of factor VIII, reported in several studies as the major cause of morbidity and mortality in COVID-19 patients [27].

To test the hypothesis that SARS-CoV-2 infection may affect the lingual tissues, this study investigated (i) the presence of SARS-CoV-2 inside the lingual tissues employing Real-Time RT-PCR, (ii) the possible anatomic modifications of the lingual papillae through histology, and (iii) the hypothesis of inflammatory mediated damage utilizing immunohistochemistry for ACE2, IFN-γ and factor VIII.

## 2. Materials and Methods

The present study was performed on cadavers scheduled for autopsies required by Italian law, to define the subjects’ cause of death. All the observations were, therefore, carried out on tissues that had already been dissected for autopsy purposes, in compliance with Italian regulations. The biopsies were harvested from tissues included in the standard functional dissection procedure, were minimal in number and dimensions, and performed in such a way that the esthetic features of the cadaver could be maintained, as requested by the law (art. 37 of Regolamento Polizia Mortuaria). Concerning privacy issues, an anonymization procedure was applied, which makes it impossible to trace the identity of the corpses.

Sixteen cadavers were collected between November 2020 and March 2021 at the Institute of Forensic Medicine of Milan. The Institute provided for each cadaver, the cause of death, while medical anamnesis referred to the patients’ state just before death, when available (Table 1). All bodies were placed at freezing temperature of −3 °C from the day of discovery/death up to 1–2 days before the autopsy. Defrosting was performed at a temperature of 15 °C for about 24 h. The subjects were classified according to their COVID-19 status at discovery/death as COVID-19 positive (COVID-19+) and COVID-19 negative (COVID-19−). All cadavers that resulted positive to a nasopharyngeal swab or to an immune test after death were included in the COVID-19+ group. The study followed the Italian general rules used for scientific research purposes, the Italian Mortuary Police Regulation procedures and Italian law.

### 2.1. Sample Collection

**Figure 1:**
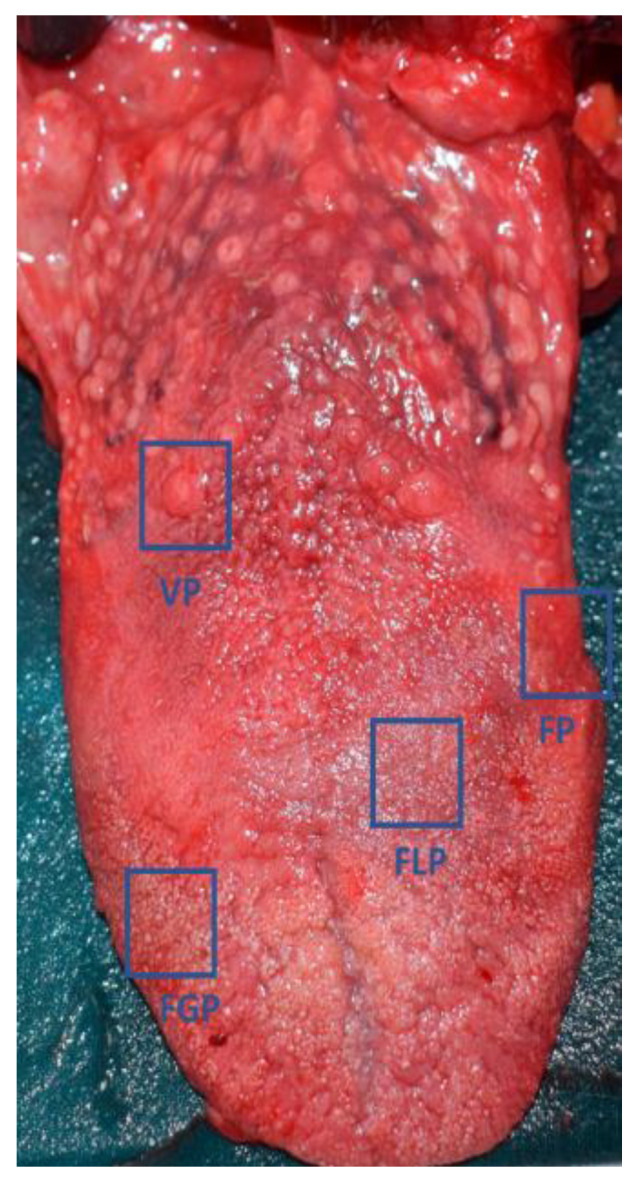
Picture of a human tongue from a cadaver. VP: circumvallate papillae; FP: foliate papillae; FLP: filiform papillae; FGP: fungiform papillae.

The tongue of the 16 subjects was macroscopically examined to identify VP, FP, FGP, and FLP, and to evaluate the possible macroscopic alteration of the gustatory and non-gustatory papillae in COVID-19+ and COVID-19− subjects. In particular, the number and size of the VP for each tongue were measured, as VP are easy to identify and susceptible to alterations [28]. The examiner who performed the macroscopic analysis (DH) was blind concerning the groups (Figure 1).

Cylindrical biopsies of the papillae were performed using a 4–6 mm punch to achieve a minimally invasive sampling (Biopsy Punch, Kai medical, 3-9-5 Iwamoto-cho, Chiyoda-ku, Tokyo 101-8586, Japan). The obtained samples were then halved longitudinally, and the first fragment was destined for histological and immunohistochemical analysis while the second one was destined for molecular analysis for SARS-CoV-2 extraction.

The halves destined to histology were fixed in 10% formalin/0.1 M phosphate buffer saline (PBS) (pH 7.4) at room temperature and the second halves were fixed in RNAlater^®^ (Qiagen, Düsseldorf, German).

### 2.2. Specimen Processing

#### 2.2.1. Molecular Processing

After RNAlater solution removal, the samples were cut into small pieces using a n° 21 blade, and about 100 µg of the sample were treated with 20 µL of Proteinase K 800 U/mL (New England Biolabs, 240 County Road, Ipswich, MA 01938-2723, USA) and 500 µL of phosphate buffer saline w/o Ca++/Mg++ (Promo Cell), vortexed, and then incubated overnight, at 56 °C 800 rpm, to obtain protein degradation. Nucleic acids were extracted from the supernatant obtained with centrifugation at 1200× *g* × 5′, using the NucliSENS^®^ easyMAG™ automated platform (BioMérieuxbioMérieux bv, Lyon, France, Benelux B.V.) in accordance with the specific B2.0 protocol with off-board lysis, not diluted silica, and a final elution volume of 55 µL [29]. Extraction quality was assessed by the amplification of the human RNase P gene and adequate samples were tested for SARS-CoV-2 by Real-Time RT-PCR according to the diagnostic protocol of the CDC targeting N1 and N2 regions [CDC. Research Use Only 2019-Novel Coronavirus (2019-nCoV) Real-time PCR Primers and Probes. Available from: https://www.cdc.gov/coronavirus/2019-ncov/lab/rt-pcr-panel-primer-probes.html] (accessed on 2 April 2022). In each Real-Time RT-PCR test, negative (DNase and RNase free water) and positive (2019-nCoV_N Positive Control, IDT) controls were included

#### 2.2.2. Histological Processing and Morphological Analysis

Samples were fixed, dehydrated in increasing concentration of ethanol (from 70% to 100%), clarified in xylol, and embedded in paraffin. From each specimen FP, VP, FLP were collected, for a total of 48 samples. FGP were not identified in any of the cadavers. Serial 6 µm sections were obtained to locate TB position and then mounted on 3-aminopropyl-trietoxi-xilane coated slides. Sections were then hydrated in decreasing concentrations of xylol and ethanol. Hematoxylin and eosin (HE) and silver impregnation (SI) (Bielschowsky method modified by Gless-Marsland) staining were performed to evaluate the morphology of lingual papillae and TB and the nervous fiber network respectively. Further, the sections were immunostained with ACE2 (BIOSS, code bs-1004R), INF-γ (RD, clone 25718) and factor VIII (DAKO, code GA527) antibodies [30]. The recovery of the antigen was performed with Proteinase K solution at 37 °C in a humidified chamber for 20 min. Slides were incubated for 50 min at room temperature with each of the antibodies. The sections were washed with PBS solution three times for 5 min, treated with the polymer labelling kit (Ultravision Quanto DetectionSystem HRP, Thermo Scientific, Loughborough, Leicestershire, UK), and revealed with diaminobenzidine (Ultravision DAB, ThermoScientific) [31]. The sections were counterstained with Mayer’s hematoxylin, dehydrated, cover-slipped, and captured by a high-resolution digital scanner (Aperio Scan Scope System CS2, Leica Biosystem, Buccinasco, Italy). Digital slides were navigated from 50× to 400× magnification.

## 3. Results

### 3.1. Study Population

The present study analyzed 8 COVID-19+ and 8 COVID-19− tongues, defined by means of nasopharyngeal swab and immune test. The COVID-19+ group consisted of 8 male individuals and the negative group included 6 male and 2 female subjects. The mean age was 52.75 ± 14.81 years for COVID-19+ and 68.00 ± 11.85 years for COVID-19−. All cadavers were stored in a deep freezer pending the autopsy. The mean time lapse between death and autopsy date was 16.75 days in COVID-19+ and 9.75 days in COVID-19—group (*t*-test: *p* = 0.048).

### 3.2. COVID-19+

#### 3.2.1. Molecular Analysis

Nucleic acids were successfully extracted from all the samples. Real-Time RT-PCR test revealed the presence of SARS-CoV-2 RNA in FLP, VP, and FP in 6 subjects out of 8 diagnosed as positive (Table 2).

#### 3.2.2. Morphological Analysis

##### Macroscopic Analysis

The anatomical structure of the tongue was well preserved in 7 out of 8 samples in the COVID-19+ group. The tongue of subject ID1, who died in a traffic accident, was strongly traumatized and did not allow a proper morphological assessment (Table 1 and Table 2, subject ID 1). The examination of the tongues revealed great interindividual variability in terms of morphology, number, and the dimension of VP. The COVID-19+ group showed an average number of 6.85 ± 1.06 VP with an average dimension of 4.28 ± 1.11 mm (Table 2).

##### Microscopic Analysis

**Figure 2:**
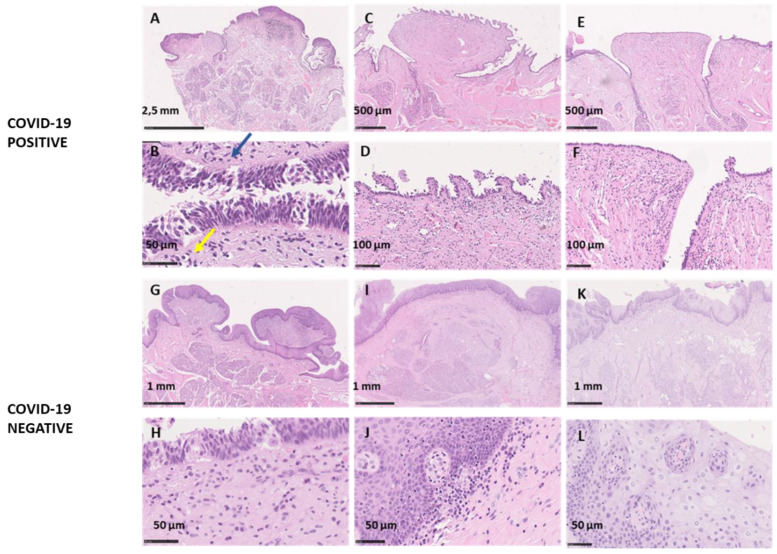
Histological sections of COVID-19+ and COVID-19− VP, HE staining for morphological evaluation. The first and third rows, namely images (**A**,**C**,**E**,**G**,**I**,**K**)**,** are overviews, while the second and fourth rows, namely images (**B**,**D**,**F**,**H**,**J**,**L**), represent their respective details at different magnifications; (**A**) overview of a COVID-19+ papilla with an apparently normal morphology; (**B**) magnification of the vallum shows TB disengaged from the epithelium (blue arrow) and a connective tissue infiltrate including cells with fibroblastic morphology (yellow arrow); (**C**) overview of a papilla with superficial extroflections; (**D**) higher magnification shows de-epithelialization of the papilla with a dense inflammatory infiltrate and fibrosis; (**E**,**F**) papilla in which some portions of the epithelial layers are absent; (**G**–**K**) overview of a COVID-19− papilla with preserved architecture; (**H**–**L**) a higher magnification shows a physiological TB aspect.

Figure 2 illustrates in a panel the morphological features of VP and FP in the COVID-19+ and COVID-19− groups. All COVID-19+ papillae showed inflammatory infiltrate and fibrosis. The greatest alteration was observed in VP. The analysis of HE stained VP at low magnification revealed that tissue integrity and structure were maintained. The structure of the papillae was preserved, and the continuity of the vallum with the Von Ebner Gland was appreciable (Figure 2A). At higher magnification (200×) no dysplastic changes were detected (Figure 2F). The superficial epithelial layer of VP seemed to be disengaged in 62% of the cases (5 out of 8) from the basement membrane (Figure 2B), with epithelial thinning in some areas of the papilla while other areas showed just a single layer of basal cells or even a complete loss of the epithelium (12%, 1 out of 8) (Figure 2D,F). At 400× magnification of papilla vallum, where the epithelial layer was still present, the epithelial structure of the buds seemed to be damaged, the typical onion shape was not preserved, and the pore was not appreciable (Figure 2B). The HE staining revealed also the presence of stromal fibrosis and inflammatory infiltrate characterized by elongated cells with a fibroblastic morphology in the sub-epithelial layer and a lymphoid patch rich in lymphocytes deeply in the center of the VP (100%, 8 out of 8) (Figure 2A,B). In one case (subject ID 5, Figure 2C,D), the VP showed epithelial-connective protrusions in the whole profile of the papilla.

**Figure 3:**
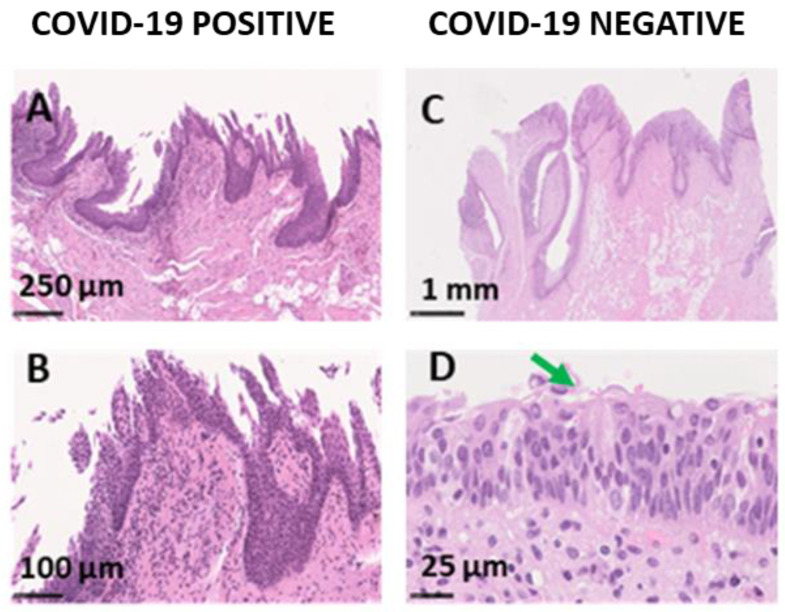
Histological sections of FP, HE staining. (**A**) COVID-19+ with a normal structure; (**B**) higher magnification shows a dense inflammatory infiltrate and fibrosis; (**C**) overview of COVID-19− papilla; (**D**) a higher magnification shows a TB, the green arrow points at the taste pit.

FP showed a preserved architecture, at high magnification, a very dense inflammatory infiltrate with stromal fibrosis was visible (Figure 3).

**Figure 4:**
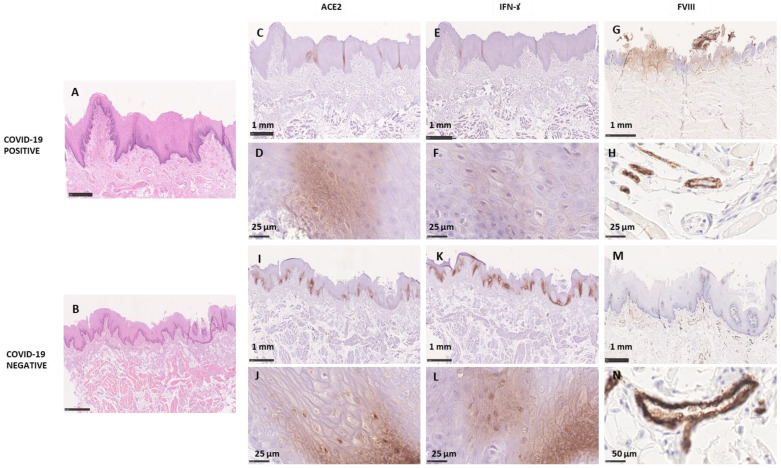
This panel shows histological and immunohistochemical sections of a COVID-19+ papilla in the upper panel, (**A**,**C**–**H**) and of a COVID-19− papilla in the lower panel, (**B**,**I**–**N**). (**A**–**C**,**E**,**G**,**I**,**K**,**M**): overviews of the papillae; (**D**,**F**,**H**,**J**,**L**,**N**): high magnification of the immunohistochemical staining.

**Figure 5:**
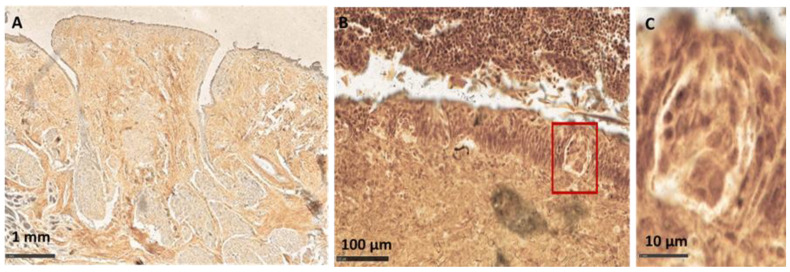
Evaluation of nerve fibers networks in VP and FP, staining with silver impregnation (Bielschowsky method modified by Gless-Marsland). (**A**) Overview of COVID-19+ VP; (**B**,**C**) details of TB in COVID-19− FP ((**C**) is a magnification of the area in the rectangle of (**B**)).

FLP showed a well-preserved structure in all samples, no signs of disaggregation of the layers were observed, the epithelium and the connective tissue did not show signs of alteration of the microstructure of the papilla (Figure 4). Silver impregnation showed no signs of alteration of the nervous fibers (Figure 5).

The immunostaining showed the expression of ACE2 (Figure 4) in the nuclei and cytoplasm of the spinous and basal cells in a punctuate pattern in the COVID-19+ tissues. ACE2 signal was largely observed in COVID-19+ samples in the cells around blood vessels and the duct cells of Von Ebner glands. ACE2 expression was also appreciable in TB cells. The expression of IFN-γ (Figure 4) was comparable to ACE2 concerning the localization and the intensity of the immunohistochemical signal (Figure 4). A strong staining response for factor VIII antibody was observed in the cytoplasm of the endothelial cells in all the specimens. A pronounced reaction was evident in both small and large vessels as well as in the plasma contained in the vessels.

### 3.3. COVID-19−

#### 3.3.1. Molecular Analysis

All the samples were adequate to the Real-Time RT-PCR analysis and tested negative for SARS-CoV-2 RNA.

#### 3.3.2. Morphological Analysis

##### Macroscopic Analysis

All COVID-19− samples showed a well-preserved anatomical structure. The macroanalysis of VP evidenced an average number of 8.12 ± 1.55 papillae with a dimension of 3.87 ± 0.99 mm (Table 1 and Table 2).

##### Microscopic Analysis

COVID-19− samples evidenced a low degree of inflammation. The analyzed gustatory papillae, VP and FP, maintained their architecture with no signs of dysplasia or any alteration. TB were appreciable, maintaining the typical bulb shape apically converging in the taste pit (Figure 2). The SI staining showed no signs of nervous fibers alteration for COVID-19− samples (Figure 3). COVID-19− samples showed intense staining for ACE2 and IFN-γ expressed in the same area as COVID-19+ samples, in the granulosum and basal layer of the epithelium (Figure 4). Factor VIII expression was comparable to COVID-19+ samples (Figure 4).

## 4. Discussion

This preliminary study aimed to verify the presence of SARS-CoV-2 in the lingual tissues of cadavers resulted positive to COVID-19 and to describe their histological and immunohistochemical gustatory and non-gustatory papillae’s features.

The cadavers classified as COVID-19+ resulted positive to nasopharyngeal swab or to immune test; therefore, the COVID-19+ group consisted of subjects who had resulted positive to SARS-CoV-2 at the time of death or had been positive to the virus in the previous months.

Previous studies already reported the presence of the RNA of SARS-CoV-2 in living subjects from formalin-fixed paraffin-embedded lingual and gland tissues [32]. Our work evidenced the presence of SARS-CoV-2 RNA in lingual samples obtained from COVID-19+ cadavers and preserved in RNAlater solution, highlighting the availability of SARS-CoV-2 in the oral tissues also after death.

Six out of eight COVID-19+ samples resulted positive to SARS-CoV-2 Real-Time RT-PCR analysis. One of the two negative samples belonged to subject ID1, a man who died following traumatic injuries and whose tongue could, therefore, not be morphologically analyzed. This subject’s COVID-19 status was casually revealed during routine hospital tests but was not anticipated by any COVID-19 symptomatology. Such finding could suggest the hypothesis that the virus might not harbor the oral tissues since the very early phases of the disease. As a matter of fact, the penetration capacity of the virus inside the tissues in asymptomatic subjects or in the early stages of the disease is very controversial [33], despite SARS-CoV-2 can be early detected in the saliva [18,19]. The other negative sample belonged to subject ID3 whose anamnestic data were unknown and whose COVID-19 diagnosis followed an immune test that resulted positive for IgG. Therefore, this subject might have been COVID-19− at the time of death. The VP of subject ID3 were characterized by a very thin layer of epithelial cells, absent in some portions of the papilla (Figure 2E,F and Figure 3A), with an infiltrate of fibroblastic-like cells, signs that could suggest a chronic damage of the papillae.

The morphological evaluation of the tongues evidenced no macroscopic differences between the two groups. The microscopic observation showed a high degree of inflammation and destruction of TB with the disengagement of the superficial epithelial layers in COVID-19+ papillae, suggesting that SARS-CoV-2 could have induced an inflammatory damage to the sensory cells of TB. Some authors have reported that SARS-CoV-2 may compromise the infected cells by viral distribution within the cytoplasm and with consequential and extensive vacuolization and apoptosis, in an ex vivo study on airway epithelium organotypic culture and cadaver [34,35]. SARS-CoV-2 could damage in the same way the gustatory sensory cells, namely through viral replication and subsequent inflammation and the destruction of TB. TB damage can cause gustatory impairment with recovery times that vary among patients. Ninety percent of the patients regain taste function in 4–15 days, a time lapse compatible with the life span of the gustatory sensory cells (15–20 days) [36]. Longer recovery times may be due to the neuroinvasive or neurotropic properties of SARS-COVID-2 or to impaired taste receptor stem cells activity [10,22,37].

In the present study, the presence of ACE2 was observed in the nuclei and cytoplasm of the stratum granulosum of the lingual tissues. It was also detected, by Sakaguchi et al. and Sawa et al., in the healthy mucosa of the keratinized stratified squamous epithelia of the tongue and non-keratinized stratified squamous epithelia of the lip and cheek, in the cytoplasm and on the cell membrane, mainly in the stratum granulosum [38,39]. Okada et al., 2021, reported that gustatory and non-gustatory lingual papillae and gustatory sensory cells also express ACE2 [10]. In the present study, we observed a downregulated expression of ACE2 in COVID-19+ tissues compared to COVID-19− ones: the ACE2 signal was observed in a punctuate pattern in COVID-19+ samples while it was denser in COVID-199− papillae. Several studies have demonstrated that SARS-CoV infection can downregulate ACE2 expression on cells causing severe organ injury, in the pulmonary tissues [40,41,42,43]. Our findings seem to confirm that SARS-CoV-2 might have the same effect on the tongue.

In our study, IFN-γ was observed in the stratum granulosum of the lingual papillae. IFN-γ was less expressed in COVID-19+ tissues than in COVID-19− ones, with a similar distribution as ACE2, suggesting that IFN-γ could be strictly related to ACE-2, whose receptor is an interferon-stimulated gene, as reported by Ziegler et al. [44,45]. IFN-γ is an antimicrobial cytokine whose signaling has been demonstrated to be increased following SARS-CoV-2 infection, locally and systemically, and seems to play a crucial role in the response to infection-induced inflammation also in TB [42,43,46]. In a study in mice, the authors reported that mimicking bacterial and viral infections altered gene expression patterns in TB cells. When either IFN-α or IFN-γ were systemically administered, a significant increase in the number of TB cells undergoing programmed cell death was observed. The authors concluded that “bacterial and viral infection-induced IFNs can act directly on taste bud cells, affecting their cellular function in taste transduction, and that IFN-induced apoptosis in taste buds may cause abnormal cell turnover and skew the representation of different taste bud cell types, leading to the development of taste disorders” [47]. In general, taste dysfunctions in patients with viral and bacterial infections or other inflammatory illnesses seem to be related to infection-induced inflammation, which can affect TB through cytokine signaling pathways.

As far as it concerns factor VIII, the immunostaining evidenced no differences between COVID-19+ and COVID-19− samples. Thrombo-inflammation and hypercoagulability have been reported in several studies to be the major cause of morbidity and mortality in patients with COVID-19 [27,48]. SARS-CoV-2 acts directly and indirectly on platelets and endothelium causing inflammation and coagulopathy leading to increased thrombus formation, emboli, and hemorrhage [27]. Wichmann et al. evidenced massive pulmonary embolism arising from lower extremities deep vein thrombi as the direct cause of death in 4 out of 12 COVID-19+ autopsies [49]. Levels of prothrombotic acute phase reactants, such as fibrinogen, von Willebrand factor, and factor VIII, that are commonly elevated during inflammatory conditions, have been reported to be increased in patients with COVID-19 compared to healthy individuals, implicating the endothelium, platelets, and the coagulation system as potential mediators of coagulopathy and thrombosis in COVID-19. However, factor VIII levels were demonstrated to be higher in blood samples of patients with COVID-19 [27]. In our study, the distribution of factor VIII in the lingual tissue was similar in COVID-19+ and COVID-19− samples. Other authors analyzed its presence in the lung tissue of COVID-19+ and COVID-199− cadavers and observed a greater expression in COVID-19+ samples [50]. However, the pulmonary endothelium produces locally factor VIII, as it is an extrahepatic site of its production, and this might explain the difference between results [51].

This article provides one of the first reports about the morphological, immunohistochemical, and molecular features of the lingual papillae in COVID-19+ cadavers compared to COVID-19−. Limitations of this study are the small sample size and a limited knowledge of the subjects’ medical history. For example, it is not known whether COVID-19+ subjects had gustatory disfunctions prior to death, or if they were on any medications that could alter or influence their inflammatory response. Such limitations prompt for further research before assuming a connection between our findings and taste alterations. The COVID-19+ group showed a longer time lapse between death time and autopsies/biopsies compared to the negative group, with a significant *p*-value. However, since cadaver storage by deep-freezing allows proper preservation of the samples and the time lapse that occurred between the discovery/death and the freezing lasted maximum 2 days, this time difference could have hardly affected the quality and characteristics of the samples [52,53].

## 5. Conclusions

In conclusion, in our study, we evidenced an inflammatory damage to the lingual papillae, probably mediated by ACE2 and IFN-γ, in COVID-19+ cadavers. Further investigations are needed in order to confirm these findings and to deepen the association between taste disorders and inflammation following SARS-CoV-2 infection.

## Figures and Tables

**Table 1:** Characterization of the studied population.

Subject ID	Age (Years)	Gender	Death-Autopsy Time Lapse (Days)	Pre-Death State	Cause of Death
COVID-19 positive					
1	41	M	9	Unknown	Traffic accident
2	65	M	17	Influenza-like symptoms	Bilateral pneumonia
3	67	M	7	Unknown	Cardiac arrest
4	73	M	7	Previous hospitalization for COVID-19 related respiratory symptoms	Cardiac arrest
5	57	M	57	Influenza-like symptoms	Pulmonary edema
6	47	M	10	Influenza-like symptoms	Pulmonary edema
7	25	M	11	Influenza-like symptoms	Cardiac arrest
8	47	M	16	Influenza-like symptoms	Cardiac arrest
COVID-19 negative					
9	68	M	6	Influenza-like symptoms in HIV+	Cardiac arrest
10	50	M	8	Influenza-like symptoms	Cardiac arrest
11	85	M	5	Unknown	Stroke
12	77	F	30	Unknown	Cardiac arrest
13	51	M	8	Influenza-like symptoms	Cardiac arrest
14	72	M	11	Unknown	Cardiac arrest
15	63	F	3	Unknown	Trauma
16	78	M	7	Unknown	Cardiac Arrest

**Table 2:** Molecular and macroscopic results of the analyzed papillae.

Subject ID	Real-Time RT-PCR	VP (*n*)	VP Dimension (mm)
COVID-19 positive			
1	Negative	/	/
2	Positive	8	4–5
3	Negative	7	3
4	Positive	7	6
5	Positive	6	5
6	Positive	8	4
7	Negative	9	2–3
8	Negative	8	4–5
COVID-19 negative			
9	Negative	11	2–6
10	Negative	6	3
11	Negative	7	4
12	Negative	9	3–4
13	Negative	8	4
14	Negative	7	4
15	Negative	9	2–3
16	Negative	8	4–5

## Data Availability

The data that support the findings of this study are available from the corresponding author upon reasonable request.

## References

[1] Bax F., Tascini C., Valente M., Marini A., Surcinelli A., Pellitteri G., De Carlo C., Gerussi V., Gigli G.L. (2020). Hyposmia and Dysgeusia in COVID-19. Neurol. Clin. Pract..

[2] Mutiawati E., Fahriani M., Mamada S.S., Fajar J.K., Frediansyah A., Maliga H.A., Ilmawan M., Bin Emran T., Ophinni Y., Ichsan I. (2021). Anosmia and dysgeusia in SARS-CoV-2 infection: Incidence and effects on COVID-19 severity and mortality, and the possible pathobiology mechanisms—A systematic review and meta-analysis. F1000Research.

[3] Nouchi A., Chastang J., Miyara M., Lejeune J., Soares A., Ibanez G., Saadoun D., Morélot-Panzini C., Similowski T., Amoura Z. (2021). Prevalence of hyposmia and hypogeusia in 390 COVID-19 hospitalized patients and outpatients: A cross-sectional study. Eur. J. Clin. Microbiol..

[4] Wu F., Zhao S., Yu B., Chen Y.-M., Wang W., Song Z.-G., Hu Y., Tao Z.-W., Tian J.-H., Pei Y.-Y. (2020). A new coronavirus associated with human respiratory disease in China. Nature.

[5] Zahra S.A., Iddawela S., Pillai K., Choudhury R.Y., Harky A. (2020). Can symptoms of anosmia and dysgeusia be diagnostic for COVID-19?. Brain Behav..

[6] Afshar Z.M., Barary M., Ebrahimpour S., Janbakhsh A., Afsharian M., Hasanpour A., Babazadeh A. (2022). Pathophysiology and Management of Tongue Involvement in COVID-19 Patients. Indian J. Otolaryngol. Head Neck Surg..

[7] Chi A.C., Neville B.W., Krayer J.W., Gonsalves W.C. (2010). Oral Manifestations of Systemic Disease. Am. Fam. Physician.

[8] Stone L.M., Tan S.-S., Tam P.P.L., Finger T. (2002). Analysis of Cell Lineage Relationships in Taste Buds. J. Neurosci..

[9] Suzuki T. (2007). Cellular Mechanisms in Taste Buds. Bull. Tokyo Dent. Coll..

[10] Okada Y., Yoshimura K., Toya S., Tsuchimochi M. (2021). Pathogenesis of taste impairment and salivary dysfunction in COVID-19 patients. Jpn. Dent. Sci. Rev..

[11] Vaira L.A., Salzano G., Fois A.G., Piombino P., De Riu G. (2020). Potential pathogenesis of ageusia and anosmia in COVID-19 patients. Int. Forum Allergy Rhinol..

[12] Kirschenbaum D., Imbach L.L., Ulrich S., Rushing E.J., Keller E., Reimann R.R., Frauenknecht K., Lichtblau M., Witt M., Hummel T. (2020). Inflammatory olfactory neuropathy in two patients with COVID-19. Lancet.

[13] Vaira L.A., Hopkins C., Sandison A., Manca A., Machouchas N., Turilli D., Lechien J.R., Barillari M.R., Salzano G., Cossu A. (2020). Olfactory epithelium histopathological findings in long-term coronavirus disease 2019 related anosmia. J. Laryngol. Otol..

[14] Khan M., Yoo S.-J., Clijsters M., Backaert W., Vanstapel A., Speleman K., Lietaer C., Choi S., Hether T.D., Marcelis L. (2021). Visualizing in deceased COVID-19 patients how SARS-CoV-2 attacks the respiratory and olfactory mucosae but spares the olfactory bulb. Cell.

[15] Maiorano E., Calastri A., Robotti C., Cassaniti I., Baldanti F., Zuccaro V., Stellin E., Ferretti V.V., Klersy C., Benazzo M. (2021). Clinical, virological and immunological evolution of the olfactory and gustatory dysfunction in COVID-19. Am. J. Otolaryngol..

[16] Hannum M.E., Koch R.J., Ramirez V.A., Marks S.S., Toskala A.K., Herriman R.D., Lin C., Joseph P.V., Reed D.R. (2022). Taste loss as a distinct symptom of COVID-19: A systematic review and meta-analysis. Chem. Senses.

[17] Vaira L.A., Lechien J.R., Salzano G., Salzano F.A., Maglitto F., Saussez S., De Riu G. (2020). Gustatory Dysfunction: A Highly Specific and Smell-Independent Symptom of COVID-19. Indian J. Otolaryngol. Head Neck Surg..

[18] Borghi E., Massa V., Carmagnola D., Dellavia C., Parodi C., Ottaviano E., Sangiorgio A., Barcellini L., Gambacorta G., Forlanini F. (2021). Saliva Sampling for Chasing SARS-CoV-2: A Game-Changing Strategy. Pharmacol. Res..

[19] Carmagnola D., Pellegrini G., Canciani E., Henin D., Perrotta M., Forlanini F., Barcellini L., Dellavia C. (2021). Saliva Molecular Testing for SARS-CoV-2 Surveillance in Two Italian Primary Schools. Children.

[20] Varga Z., Flammer A.J., Steiger P., Haberecker M., Andermatt R., Zinkernagel A.S., Mehra M.R., Schuepbach R.A., Ruschitzka F., Moch H. (2020). Endothelial cell infection and endotheliitis in COVID-19. Lancet.

[21] Sato T., Ueha R., Goto T., Yamauchi A., Kondo K., Yamasoba T. (2021). Expression of ACE2 and TMPRSS2 Proteins in the Upper and Lower Aerodigestive Tracts of Rats: Implications on COVID 19 Infections. Laryngoscope.

[22] Doyle M.E., Appleton A., Liu Q.-R., Yao Q., Mazucanti C.H., Egan J.M. (2021). Human Type II Taste Cells Express Angiotensin-Converting Enzyme 2 and Are Infected by Severe Acute Respiratory Syndrome Coronavirus 2 (SARS-CoV-2). Am. J. Pathol..

[23] Costela-Ruiz V.J., Illescas-Montes R., Puerta-Puerta J.M., Ruiz C., Melguizo-Rodríguez L. (2020). SARS-CoV-2 infection: The role of cytokines in COVID-19 disease. Cytokine Growth Factor Rev..

[24] Karki R., Sharma B.R., Tuladhar S., Williams E.P., Zalduondo L., Samir P., Zheng M., Sundaram B., Banoth B., Malireddi R.K.S. (2021). Synergism of TNF-α and IFN-γ Triggers Inflammatory Cell Death, Tissue Damage, and Mortality in SARS-CoV-2 Infection and Cytokine Shock Syndromes. Cell.

[25] Yamashita H., Nakagawa K., Tago M., Nakamura N., Shiraishi K., Eda M., Nakata H., Ms N.N., Yokoyama R., Bs M.O. (2006). Taste dysfunction in patients receiving radiotherapy. Head Neck.

[26] Henkin R.I., Larson A.L., Powell R.D. (1975). Hypogeusia, Dysgeusia, Hyposmia, and Dysosmia following Influenza-Like Infection. Ann. Otol. Rhinol. Laryngol..

[27] Gu S.X., Tyagi T., Jain K., Gu V.W., Lee S.H., Hwa J.M., Kwan J.M., Krause D.S., Lee A.I., Halene S. (2021). Thrombocytopathy and Endotheliopathy: Crucial Contributors to COVID-19 Thromboinflammation. Nat. Rev. Cardiol..

[28] Arey L.B., Tremaine M.J., Monzingo F.L. (1935). The numerical and topographical relations of taste buds to human circumv allate papillae throughout the life span. Anat. Rec..

[29] Frati E., Bianchi S., Amendola A., Colzani D., Petrelli F., Zehender G., Tanzi E. (2019). Genetic characterization of variants of HPV-16, HPV-18 and HPV-52 circulating in Italy among general and high-risk populations. Mol. Med. Rep..

[30] Croci G., Vaira V., Trabattoni D., Biasin M., Valenti L., Baselli G., Barberis M., Rocco E.G., Gregato G., Scandroglio M. (2021). Emergency Lung Transplantation after COVID-19: Immunopathological Insights on Two Affected Patients. Cells.

[31] Dellavia C., Canullo L., Allievi C., Lang N.P., Pellegrini G. (2013). Soft tissue surrounding switched platform implants: An immunohistochemical evaluation. Clin. Oral Implant. Res..

[32] Guerini-Rocco E., Taormina S.V., Vacirca D., Ranghiero A., Rappa A., Fumagalli C., Maffini F., Rampinelli C., Galetta D., Tagliabue M. (2020). SARS-CoV-2 detection in formalin-fixed paraffin-embedded tissue specimens from surgical resection of tongue squamous cell carcinoma. J. Clin. Pathol..

[33] Bradley B.T., Maioli H., Johnston R., Chaudhry I., Fink S.L., Xu H., Najafian B., Deutsch G., Lacy J.M., Williams T. (2020). Histopathology and ultrastructural findings of fatal COVID-19 infections in Washington State: A case series. Lancet.

[34] Zhu N., Zhang D., Wang W., Li X., Yang B., Song J., Zhao X., Huang B., Shi W., Lu R. (2020). A Novel Coronavirus from Patients with Pneumonia in China, 2019. N. Engl. J. Med..

[35] Borczuk A.C., Salvatore S.P., Seshan S.V., Patel S.S., Bussel J.B., Mostyka M., Elsoukkary S., He B., DEL Vecchio C., Fortarezza F. (2020). COVID-19 pulmonary pathology: A multi-institutional autopsy cohort from Italy and New York City. Mod. Pathol..

[36] Witt M. (2019). Anatomy and development of the human taste system. Neurogenetics, Part II.

[37] Meinhardt J., Radke J., Dittmayer C., Franz J., Thomas C., Mothes R., Laue M., Schneider J., Brünink S., Greuel S. (2021). Olfactory transmucosal SARS-CoV-2 invasion as a port of central nervous system entry in individuals with COVID-19. Nat. Neurosci..

[38] Sakaguchi W., Kubota N., Shimizu T., Saruta J., Fuchida S., Kawata A., Yamamoto Y., Sugimoto M., Yakeishi M., Tsukinoki K. (2020). Existence of SARS-CoV-2 Entry Molecules in the Oral Cavity. Int. J. Mol. Sci..

[39] Sawa Y., Ibaragi S., Okui T., Yamashita J., Ikebe T., Harada H. (2021). Expression of SARS-CoV-2 entry factors in human oral tissue. J. Anat..

[40] Haga S., Yamamoto N., Nakai-Murakami C., Osawa Y., Tokunaga K., Sata T., Sasazuki T., Ishizaka Y. (2008). Modulation of TNF- -converting enzyme by the spike protein of SARS-CoV and ACE2 induces TNF- production and facilitates viral entry. Proc. Natl. Acad. Sci. USA.

[41] Glowacka I., Bertram S., Herzog P., Pfefferle S., Steffen I., Muench M.O., Simmons G., Hofmann H., Kuri T., Weber F. (2010). Differential Downregulation of ACE2 by the Spike Proteins of Severe Acute Respiratory Syndrome Coronavirus and Human Coronavirus NL63. J. Virol..

[42] Kuba K., Imai Y., Rao S., Gao H., Guo F., Guan B., Huan Y., Yang P., Zhang Y., Deng W. (2005). A crucial role of angiotensin converting enzyme 2 (ACE2) in SARS coronavirus–induced lung injury. Nat. Med..

[43] Oudit G.Y., Kassiri Z., Jiang C., Liu P.P., Poutanen S., Penninger J., Butany J. (2009). SARS-coronavirus modulation of myocardial ACE2 expression and inflammation in patients with SARS. Eur. J. Clin. Investig..

[44] Ziegler C.G.K., Allon S.J., Nyquist S.K., Mbano I.M., Miao V.N., Tzouanas C.N., Cao Y., Yousif A.S., Bals J., Hauser B.M. (2020). SARS-CoV-2 Receptor ACE2 Is an Interferon-Stimulated Gene in Human Airway Epithelial Cells and Is Detected in Specific Cell Subsets across Tissues. Cell.

[45] Heuberger J., Trimpert J., Vladimirova D., Goosmann C., Lin M., Schmuck R., Mollenkopf H., Brinkmann V., Tacke F., Osterrieder N. (2021). Epithelial response to IFN-γ promotes SARS-CoV-2 infection. EMBO Mol. Med..

[46] Huang C., Wang Y., Li X., Ren L., Zhao J., Hu Y., Zhang L., Fan G., Xu J., Gu X. (2020). Clinical features of patients infected with 2019 novel coronavirus in Wuhan, China. Lancet.

[47] Wang H., Zhou M., Brand J., Huang L. (2007). Inflammation Activates the Interferon Signaling Pathways in Taste Bud Cells. J. Neurosci..

[48] Rapkiewicz A.V., Mai X., Carsons S.E., Pittaluga S., Kleiner D.E., Berger J.S., Thomas S., Adler N., Charytan D., Gasmi B. (2020). Megakaryocytes and platelet-fibrin thrombi characterize multi-organ thrombosis at autopsy in COVID-19: A case series. EClinicalMedicine.

[49] Wichmann D., Sperhake J.P., Lütgehetmann M., Steurer S., Edler C., Heinemann A., Heinrich F., Mushumba H., Kniep I., Schröder A.S. (2020). Autopsy Findings and Venous Thromboembolism in Patients with COVID-19. Ann. Intern. Med..

[50] Cipolloni L., Sessa F., Bertozzi G., Baldari B., Cantatore S., Testi R., D’Errico S., Di Mizio G., Asmundo A., Castorina S. (2020). Preliminary Post-Mortem COVID-19 Evidence of Endothelial Injury and Factor VIII Hyperexpression. Diagnostics.

[51] Jacquemin M., Neyrinck A., Hermanns M.I., Lavend’Homme R., Rega F., Saint-Remy J.-M., Peerlinck K., Van Raemdonck D., Kirkpatrick C.J. (2006). FVIII production by human lung microvascular endothelial cells. Blood.

[52] Brenner E. (2014). Human body preservation—Old and new techniques. J. Anat..

[53] Song Y.K., Jo D.H. (2021). Current and potential use of fresh frozen cadaver in surgical training and anatomical education. Anat. Sci. Educ..

